# Chronic Kidney Disease and Fibrosis: The Role of Uremic Retention Solutes

**DOI:** 10.3389/fmed.2015.00060

**Published:** 2015-08-31

**Authors:** Henricus A. M. Mutsaers, Elisabeth G. D. Stribos, Griet Glorieux, Raymond Vanholder, Peter Olinga

**Affiliations:** ^1^Department of Pharmaceutical Technology and Biopharmacy, University of Groningen, Groningen, Netherlands; ^2^Division of Nephrology, Department of Internal Medicine, University Medical Center Groningen, University of Groningen, Groningen, Netherlands; ^3^Renal Division, Department of Internal Medicine, Ghent University Hospital, Ghent, Belgium

**Keywords:** chronic kidney disease, renal fibrosis, cardiac fibrosis, uremic retention solutes, TGF-β, epithelial-to-mesenchymal transition

## Abstract

Chronic kidney disease (CKD) is a major global health concern, and the uremic state is highly associated with fibrogenesis in several organs and tissues. Fibrosis is characterized by excessive production and deposition of extracellular matrix proteins with a detrimental impact on organ function. Another key feature of CKD is the retention and subsequent accumulation of solutes that are normally cleared by the healthy kidney. Several of these uremic retention solutes, including indoxyl sulfate and p-cresyl sulfate, have been suggested to be CKD-specific triggers for the development and perpetuation of fibrosis. The purpose of this brief review is to gather and discuss the current body of evidence linking uremic retention solutes to the fibrotic response during CKD, with a special emphasis on the pathophysiological mechanisms in the kidney.

## Introduction

The kidneys are essential for the clearance of (metabolic) waste products. And even though the primary cause of kidney disease (either acute or chronic) is often related to direct injury, e.g., inflammatory damage in the case of glomerulonephritis and pyelonephritis, or hypoperfusion, ischemia, and toxic damage, this will ultimately result in a reduced renal function. When the kidneys fail to purify the body of metabolic end-products, a host of substances that are normally excreted into the urine are retained. This condition is called uremia, after the first recognized and most abundant retention product, urea. Many uremic retention solutes are biologically active and exert toxicity, affecting the functional capacity of almost every organ system in the body, resulting in the complex clinical picture of uremia (1). Currently, uremic retention products are most often classified according to their removal pattern by dialysis, which is, up to now, the most frequently applied method to reduce solute levels in patients with end-stage renal disease (ESRD). Three major groups are considered: (1) small water-soluble compounds, molecular weight (MW) <500 D, which are easy to remove by any type of dialysis; (2) larger middle molecules, mostly peptides, MW >500 D, which can only be cleared by dialyzers containing large pore membranes (high flux dialysis); and (3) protein-bound compounds, mostly with a low MW (<500 D), these solutes are difficult to remove by any type of dialysis, as protein binding hampers their clearance. Especially this class of retention solutes greatly contributes to comorbidities, such as organ fibrosis, observed in chronic kidney disease (CKD) patients.

## Fibrosis and Uremia: Clinical Aspects

Fibrosis is a process whereby functional tissue is replaced by connective tissue. Once this phenomenon exceeds the level of physiological repair, it will result in loss of organ architecture as well as loss of functional tissue. Causes may be local damage, e.g., trauma, infection, or ischemia, but also more diffuse conditions, like systemic inflammation. In what follows, we will summarize the clinical consequences of fibrotic changes in uremia.

## Renal Fibrosis

As stated above, kidney disease is often due to direct injury, yet in many cases this initial insult will initiate fibrogenesis, especially when regeneration as healing process is inadequate (2). The kidney is a complex organ containing a wide variety of cells that constitute the glomeruli, tubules, interstitium, and capillaries (3). And the initial site of injury determines the subsequent pathology, e.g., glomerular IgA deposition will cause glomerular fibrosis, whereas infections or proteinuria will provoke tubulointerstitial fibrosis (3). Still, irrespective of etiology, the subsequent fibrotic response will ultimately affect the functional capacities of the kidney, with uremia as one of the consequences. To cope with this problem, many strategies have been developed in hopes of slowing down or even reversing fibrogenesis (4). Although several studies have been successful at the pre-clinical level, only limited advances have been made at this time in the translation of these findings to the level of patient treatment (4). In addition, in the analysis of the urinary proteome related to CKD and CKD progression, a marked positive correlation appears with collagen or matrix protein fragments, which via a bottom-to-top approach confirms the pathophysiological role of fibrosis in the functional disturbance of kidneys and other tissues in patients with CKD (5).

## Cardiac Fibrosis

Similar to the kidney, fibrosis of the heart and its valves depends on a host of damaging processes, such as ischemia or inflammation, with heart failure as the functional resultant. Many of the factors that cause kidney failure, e.g., hypertension and diabetes, concurrently promote cardiac fibrosis (6, 7), a condition induced by uremic retention solutes as well (8, 9). In its turn, the ensuing cardiac failure causes hypoperfusion and ischemia of the kidneys, which is causative for uremia.

## The Cardio-Renal Axis

All these elements together result in a close interaction of renal and cardiac dysfunction, often termed, correctly or incorrectly, cardio-renal syndrome (10, 11). Nevertheless, there is no debate that kidney and heart dysfunction are closely intertwined, resulting in a high cardiovascular burden in renal failure patients (12, 13).

## Fibrosis of Other Organ Systems

It seems conceivable that uremia at large is a profibrotic condition, which may be detrimental for organ systems other than the kidney and heart. A study in CKD rats demonstrated the presence of fibrosis in the peritoneal membrane, an organ system not directly involved in hemodynamic homeostasis, within 6 weeks (14).

The most clinically relevant organ system next to the heart and the kidneys is the vascular bed. Vessel stiffness, a key pathophysiological feature of uremia, and at least in part the consequence of fibrosis (15), results in systolic hypertension, diastolic hypoperfusion, a diminished physiologic response to orthostasis and volume loss, and an enhanced risk of cardiovascular events such as myocardial ischemia and ischemic and hemorrhagic stroke (16).

Thus, it is clear that organ fibrosis is a key feature of CKD, yet the pathophysiological mechanisms underlying the fibrotic response during uremia remain to be fully elucidated.

## Uremic Solutes and Renal Fibrosis

Fibrosis is the end result of a complex cascade of cellular and molecular responses initiated by organ damage (3). And even though there is a range of organ-specific triggers, the fibrotic process and associated signaling pathways are highly conserved between different organs (3). Furthermore, in recent years, epithelial-to-mesenchymal transition (EMT) has emerged as a leading, yet highly debated, hypothesis for the origin of collagenous matrix-producing myofibroblasts that contribute to the fibrotic response (17–21). Renal fibrosis ends in uremia, yet uremia *per se* will also further enhance the fibrotic process, because of the direct biological effects of uremic toxins. In a recent systematic review on the toxicity of two uremic retention products, e.g., indoxyl sulfate and p-cresyl sulfate, of the 27 retrieved high-quality studies, at least five demonstrated a direct link to EMT and/or kidney fibrosis (22). Therefore, the remainder of this review will delineate the profibrotic impact of several uremic solutes on the kidney (summarized in Table 1).

**Table 1:** **Profibrotic effects of uremic solutes**.

Solute	Chemical formula	Avg. MW	TGF-β	PAI-1	COL1A1	Interstitial fibrosis	Glomerular sclerosis	EMT	Senescence	Klotho	Model(s)	Reference
Indoxyl sulfate	C_8_H_7_NO_4_S	213.2	↑	↑	↑			√	√	↓	*In vitro*/*In vivo*	(28, 33, 34, 38, 39, 42–44, 46, 47, 53, 54)
p-Cresyl sulfate	C_7_H_8_O_4_S	188.2	↑		↑			√		↓	*In vitro*/*In vivo*	(39, 54, 57)
p-Cresyl glucuronide	C_13_H_16_O_7_	284.3						√			*In vitro*	(56)
Hippuric acid	C_9_H_9_NO_3_	179.2					√				*In vivo*	(60)
Indole-3-acetic acid	C_10_H_9_NO_2_	175.2		↑		√	√				*In vitro*/*In vivo*	(28, 60)
Leptin	Protein		↑		↑						*In vitro*/*In vivo*	(66 –68)
Marino- bufagenin	C_24_H_32_O_5_	400.5			↑	√		√			*In vitro*/*In vivo*	(76)

*↑ increased expression; ↓ decrease expression; √ induced*.

*Avg. MW, average molecular weight; COL1A1, alpha-1 type I collagen; EMT, epithelial-to-mesenchymal transition; PAI-1, plasminogen activator inhibitor-1; TGF-β, transforming growth factor-β*.

## TGF-β Signaling Pathway in CKD

Transforming growth factor (TGF)-β is one of the key factors driving the fibrotic response in most organs. Binding of TGF-β to a serine-threonine kinase type II receptor results in the recruitment and phosphorylation of a type I receptor, which in turn phosphorylates SMADs thereby initiating a host of signaling cascades (3, 23). TGF-β is synthesized and secreted by inflammatory cells and a variety of effector cells, and activation of the pathway results in the formation and deposition of extracellular matrix proteins (3). In 1992, the role of TGF-β in renal fibrosis was still uncertain (24), yet in the following years more and more studies demonstrated the involvement of this factor in renal fibrogenesis (25–27). More recently, the interplay between uremic retention solutes, such as indoxyl sulfate and p-cresyl sulfate, and TGF-β has gained more scientific attention (28, 29).

## Impact of Indoxyl Sulfate on Fibrogenesis

Indoxyl sulfate is a small organic aromatic polycyclic anion derived from dietary tryptophan that has extensively been studied in conjunction with CKD-associated cardiovascular disease (22), and it is reported that this uremic solute can induce vascular calcification and correlates with coronary artery disease and mortality (30–32). Indoxyl sulfate is thought, however, to also contribute to a plethora of pathologies observed in dialysis patients, including tubulointerstitial inflammation and whole-kidney damage (22). Already in the 1990s, studies were published linking indoxyl sulfate to progression of renal disease as well as renal fibrosis (33, 34). Miyazaki et al. observed that indoxyl sulfate overload augmented the gene expression of tissue inhibitor of metalloproteinases (TIMP)-1, intercellular adhesion molecule (ICAM)-1, alpha-1 type I collagen (COL1A1), and TGF-β in the renal cortex of 5/6-nephrectomized rats (33, 34). Moreover, indoxyl sulfate stimulated the production of TGF-β by renal proximal tubular cells *in vitro* (34). Almost a decade later, it was demonstrated that exposure of HK-2 cells to indoxyl sulfate resulted in a reactive oxygen species (ROS)-mediated up-regulation of plasminogen activator inhibitor (PAI)-1 (28), a downstream signaling molecule of the TGF-β pathway associated with most aggressive kidney diseases (35–37). Furthermore, Saito et al. reported that indoxyl sulfate can increase α-smooth muscle actin (α-SMA) and TGF-β expression in HK-2 cells by activation of the (pro)renin receptor through ROS-Stat3-NF-κB signaling (38). Also in mouse renal proximal tubular cells, it was demonstrated that indoxyl sulfate activates the TGF-β pathway, as illustrated by an increased SMAD2/3 phosphorylation (39).

Although the contribution of EMT to fibrosis remains controversial, phenotypic alterations reminiscent of EMT, also referred to as epithelial phenotypic changes (EPC), might play a role in the fibrotic response as well as disease progression (40, 41). Several studies have demonstrated that indoxyl sulfate induces EMT, as demonstrated by a reduced expression of E-cadherin and zona occludens (ZO)-1, and increased expression of α-SMA in rat kidney as well as rat proximal tubular (NRK-52E) cells (42, 43). Furthermore, Sun et al. reported that treatment with indoxyl sulfate increased the expression of the EMT-associated transcription factor Snail, concurrent with an elevated expression of fibronectin and α-SMA as well as a diminished expression of E-cadherin in both mouse kidneys and murine proximal tubular cells (39). Similar effects of indoxyl sulfate have also been observed in human renal cell models (42, 44).

Renal cells can become senescent due to a variety of (stress) triggers, including aging, and these cells, while in growth arrest, can contribute to renal fibrosis by secreting profibrotic cytokines and growth factors (45). It has been demonstrated that exposure of HK-2 cells to indoxyl sulfate resulted in an increased expression of p53 and p65, and augmented β-galactosidase activity (46, 47), indicating that indoxyl sulfate induces senescence.

Lastly, the renoprotective anti-aging factor klotho, which is involved in a myriad of homeostatic processes (48–50), might mitigate renal fibrosis by suppressing TGF-β signaling and vice versa, deficient klotho expression may accelerate senescence and fibrosis (51, 52). Adijiang et al. reported that in both Dahl salt-resistant normotensive and Dahl salt-sensitive hypertensive rats, treatment with indoxyl sulfate resulted in lower gene expression of klotho (53). These findings were corroborated by the study of Sun and colleagues showing that indoxyl sulfate suppressed klotho expression in murine renal proximal tubules as well as HK-2 cells (54).

**Figure 1:**
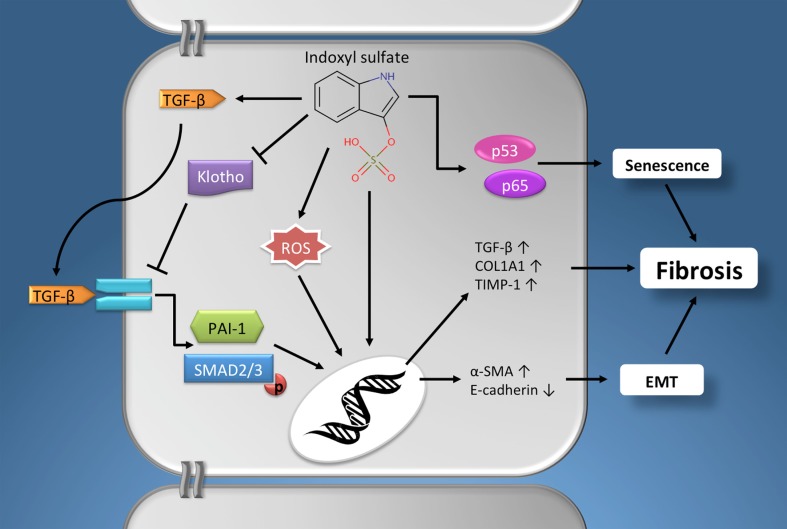
**The profibrotic effects of indoxyl sulfate**. Schematic presentation of the pathophysiological mechanisms via which indoxyl sulfate promotes fibrogenesis in renal cells. Furthermore, similar effects have been described for other protein-bound uremic retention solutes, e.g., p-cresyl sulfate. See text for details. Chemical structure obtained from the Human Metabolome Database (www.hmdb.ca). α-SMA, α-smooth muscle actin; EMT, epithelial-to-mesenchymal transition; PAI-1, plasminogen activator inhibitor-1; COL1A1, alpha-1 type I collagen; ROS, reactive oxygen species; TGF-β, Transforming growth factor-β; TIMP-1, tissue inhibitor of metalloproteinases-1.

Taken together, it is evident that indoxyl sulfate can contribute to renal fibrogenesis via an array of pathophysiological mechanisms (Figure 1), e.g., ROS production, stimulating expression of the profibrotic factor TGF-β, induction of EMT/EPC, promoting cellular senescence and by reducing klotho expression.

## Profibrotic Activity of Other Protein-Bound Solutes

Next to indoxyl sulfate, several other uremic solutes have been linked to renal fibrosis, most prominently the p-cresol metabolite, p-cresyl sulfate (22, 29). p-Cresol is formed by colonic bacteria from dietary tyrosine and this parent compound is either conjugated to sulfate or glucuronic acid giving rise to circulating p-cresyl sulfate or p-cresyl glucuronide (55). The main profibrotic effect currently described for p-cresyl sulfate is the induction of TGF-β (protein) expression. Sun et al. reported that exposure of murine renal proximal tubular cells to p-cresyl sulfate resulted in an increased expression of TGF-β and SMAD phosphorylation, concurrent with the induction of EMT (39). Conversely, in human conditionally immortalized renal proximal tubule epithelial cells, p-cresyl sulfate failed to induce EMT, whereas p-cresyl glucuronide did promote phenotypical changes associated with EMT (56). Furthermore, Watanabe et al. showed a ROS-dependent production and secretion of TGF-β protein in HK-2 cells upon treatment with p-cresyl sulfate (57). Moreover, they reported that p-cresyl sulfate increased the gene expression of TIMP-1 and COL1A1 (57). And, similar to indoxyl sulfate, it has been demonstrated that p-cresyl sulfate mitigated the expression of klotho in both murine and human renal cell models (54).

Two other widely studied uremic solutes are hippuric acid and indole-3-acetic acid. Both protein-bound compounds have deleterious effects on normal renal (metabolic) functioning (58, 59), yet there is scant evidence for their potential impact on fibrosis. Satoh and colleagues demonstrated that treatment with either hippuric acid or indole-3-acetic acid induced glomerular sclerosis in rats (60). And indole-3-acetic acid stimulated interstitial fibrosis (60). Also, it has been reported that indole-3-acetic acid activated the TGF-β pathway in HK-2 cells, as illustrated by an increased expression of PAI-1 (28).

Thus, several of the protein-bound uremic retention solutes, although chemically very diverse entities, can elicit similar toxic effects thereby promoting renal fibrosis. Yet, the majority, if not all, of this evidence has been obtained experimentally in animals or by *in vitro* studies, whereas clinical studies on this aspect are virtually absent. Therefore, far more (clinical) research is needed to fully characterize the possible profibrotic effects of the more than 150 cataloged uremic retention solutes.

## New Kids on the Block

Next to the widely studied protein-bound uremic toxins, several other lesser-known retention solutes might play a role in renal fibrosis, for instance leptin and marinobufagenin (MBG).

Leptin (from the Greek word leptos, meaning “thin”) is a product of the obese gene, identified in 1994 (61), and is secreted by adipocytes. Activation of its signaling pathway in the hypothalamus reduces food intake and increases energy expenditure. This adipocytokine is eliminated from the circulation via the kidneys mainly by metabolic degradation in the tubules (62). And it has been reported that serum leptin levels are increased in CKD and ESRD patients (63, 64). The mechanisms underlying leptin retention are complex as reviewed by Alix et al.: decreased renal clearance, increased fat mass, hyperinsulinemia, and low-grade chronic inflammation all contribute to hyperleptinemia in CKD patients (65). Furthermore, it has been reported by Wolf and colleagues that leptin plays a role in the progression of renal fibrogenesis (66). They demonstrated that leptin triggers glomerular endothelial cell proliferation via TGF-β. In addition, Briffa et al. reported that leptin increased TGF-β production and secretion in opossum kidney proximal tubule cells (67). Furthermore, it is shown that leptin stimulates COL1A1 production in renal interstitial (NRK-49F) fibroblasts (68). Moreover, hyperleptinemia is associated with increased blood pressure, a known risk factor for renal fibrosis, and additional deleterious (potential profibrotic) effects of this adipocytokine have been described in experimental and clinical studies (69, 70). Noteworthy, two classes of compounds with similar damaging effects on the cardiovascular system as leptin are dimethylarginines and advanced glycation end-products; however, more studies are needed to unveil the suspected profibrotic potential of these compounds. For an overview of the toxicity of both groups of solutes, the interested reader is referred to the reviews by Schepers et al., and Mallipattu et al. (71, 72).

Marinobufagenin belongs to the family of endogenous cardiotonic steroids (CTS), also known as digitalis-like factors, and is produced by adrenal cortical cells (73). MBG is a Na + /K + -ATPase inhibitor that specifically binds to the α subunit of the sodium pump. This results in renal sodium excretion, increased myocardial contractility, and vasoconstriction (73). Therefore, CTS derived from dried toad skins were already used 1000 years ago in traditional medicine to treat congestive heart failure. Elevated levels of MGB have been detected in CKD and hemodialysis patients (74, 75), and Fedorova et al. reported that MBG stimulated renal fibrosis in rats as well as increased tubular expression of Snail (76). Furthermore, they demonstrated that MGB induced EMT in LLC-PK1 cells as observed by increased levels of collagen I, fibronectin, and vimentin (76). In line with the observed profibrotic effect of MBG, it was reported that immunization against this steroid attenuated renal fibrosis in 5/6-nephrectomized rats (77).

These thought-provoking results warrant further scrutiny and without a doubt many more uremic retention solutes may be classified as profibrotic in the near future.

## Future Directions

CKD is a growing health concern and renal fibrosis is an integral part of the pathophysiological mechanism underlying disease progression. Current therapies for renal fibrosis mainly focus on the etiology of the disease, such as hypertension or diabetes, and as such show only limited efficacy in halting the fibrotic process (3). A key feature of uremia is the accumulation of a wide array of potential toxic solutes and slowly a body of evidence is emerging implicating these retention solutes as culprits in CKD-associated (renal) fibrogenesis. Therefore, therapies aimed at limiting the intake/absorption/production of uremic solutes, such as oral adsorbents or probiotics (78–80), or treatment modalities supporting the clearance of these compounds, e.g., living dialysis membranes (81), will most likely have a great potential for slowing fibrosis. Moreover, better understanding of the profibrotic effects of the multiplicity of uremic retention solutes will further aid in unveiling novel therapeutic targets.

## Author Contributions

HM and PO conceived the manuscript. HM, ES, and RV wrote the manuscript. GG and PO critically revised and improved the manuscript writing. All authors approved the final version of the manuscript and fully agree with its content.

## Conflict of Interest Statement

The authors declare that the writing of this review was conducted in the absence of any commercial or financial relationships that could be construed as a potential conflict of interest.

## Funding

This work was supported by the Netherlands Organisation for Health Research and Development (ZonMW; grant number 114021010).

## References

[1] Meyer TW Hostetter TH Uremia. N Engl J Med (2007) 357(13):1316–25. 10.1056/NEJMra071313 17898101

[2] Liu Y. Renal fibrosis: new insights into the pathogenesis and therapeutics. Kidney Int (2006) 69(2):213–7. 10.1038/sj.ki.5000054 16408108

[3] Rockey DC Bell PD Hill JA Fibrosis – a common pathway to organ injury and failure. N Engl J Med (2015) 372(12):1138–49. 10.1056/NEJMra1300575 25785971

[4] Lee SY Kim SI Choi ME. Therapeutic targets for treating fibrotic kidney diseases. Transl Res (2015) 165(4):512–30. 10.1016/j.trsl.2014.07.010 25176603 PMC4326607

[5] Schanstra JP Zurbig P Alkhalaf A Argiles A Bakker SJ Beige J Diagnosis and prediction of CKD progression by assessment of urinary peptides. J Am Soc Nephrol (2015) 26(8):1999–2010. 10.1681/ASN.2014050423 25589610 PMC4520165

[6] Yamazaki KG Gonzalez E Zambon AC. Crosstalk between the renin-angiotensin system and the advance glycation end product axis in the heart: role of the cardiac fibroblast. J Cardiovasc Transl Res (2012) 5(6):805–13. 10.1007/s12265-012-9405-4 23054657

[7] Gonzalez A Lopez B Diez J. Fibrosis in hypertensive heart disease: role of the renin-angiotensin-aldosterone system. Med Clin North Am (2004) 88(1):83–97. 10.1016/S0025-7125(03)00125-1 14871052

[8] Lekawanvijit S Adrahtas A Kelly DJ Kompa AR Wang BH Krum H. Does indoxyl sulfate, a uraemic toxin, have direct effects on cardiac fibroblasts and myocytes? Eur Heart J (2010) 31(14):1771–9. 10.1093/eurheartj/ehp574 20047993

[9] Yisireyili M Shimizu H Saito S Enomoto A Nishijima F Niwa T. Indoxyl sulfate promotes cardiac fibrosis with enhanced oxidative stress in hypertensive rats. Life Sci (2013) 92(24–26):1180–5. 10.1016/j.lfs.2013.05.008 23702423

[10] Ronco C Di Lullo L. Cardiorenal syndrome. Heart Fail Clin (2014) 10(2):251–80. 10.1016/j.hfc.2013.12.003 24656104

[11] Lekawanvijit S Kompa AR Wang BH Kelly DJ Krum H. Cardiorenal syndrome: the emerging role of protein-bound uremic toxins. Circ Res (2012) 111(11):1470–83. 10.1161/CIRCRESAHA.112.278457 23139286

[12] Vanholder R Massy Z Argiles A Spasovski G Verbeke F Lameire N Chronic kidney disease as cause of cardiovascular morbidity and mortality. Nephrol Dial Transplant (2005) 20(6):1048–56. 10.1093/ndt/gfh813 15814534

[13] Chronic Kidney Disease Prognosis Consortium Matsushita K van der Velde M Astor BC Woodward M Levey AS Association of estimated glomerular filtration rate and albuminuria with all-cause and cardiovascular mortality in general population cohorts: a collaborative meta-analysis. Lancet (2010) 375(9731):2073–81. 10.1016/S0140-6736(10)60674-5 20483451 PMC3993088

[14] De Vriese AS Tilton RG Mortier S Lameire NH. Myofibroblast transdifferentiation of mesothelial cells is mediated by RAGE and contributes to peritoneal fibrosis in uraemia. Nephrol Dial Transplant (2006) 21(9):2549–55. 10.1093/ndt/gfl271 16757496

[15] Verbeke F Van Biesen W Vanholder R The role of collagen metabolism in CKD-associated arterial senescence: underestimated and underappreciated. Nephrol Dial Transplant (2011) 26(9):2726–8. 10.1093/ndt/gfr421 21836167

[16] Mitchell GF Hwang SJ Vasan RS Larson MG Pencina MJ Hamburg NM Arterial stiffness and cardiovascular events: the Framingham Heart Study. Circulation (2010) 121(4):505–11. 10.1161/CIRCULATIONAHA.109.886655 20083680 PMC2836717

[17] Zeisberg M Duffield JS. Resolved: EMT produces fibroblasts in the kidney. J Am Soc Nephrol (2010) 21(8):1247–53. 10.1681/ASN.2010060616 20651165

[18] Liu Y. New insights into epithelial-mesenchymal transition in kidney fibrosis. J Am Soc Nephrol (2010) 21(2):212–22. 10.1681/ASN.2008121226 20019167 PMC4554339

[19] Kriz W Kaissling B Le Hir M. Epithelial-mesenchymal transition (EMT) in kidney fibrosis: fact or fantasy? J Clin Invest (2011) 121(2):468–74. 10.1172/JCI44595 21370523 PMC3026733

[20] Duffield JS. Cellular and molecular mechanisms in kidney fibrosis. J Clin Invest (2014) 124(6):2299–306. 10.1172/JCI72267 24892703 PMC4038570

[21] Kalluri R Neilson EG. Epithelial-mesenchymal transition and its implications for fibrosis. J Clin Invest (2003) 112(12):1776–84. 10.1172/JCI200320530 14679171 PMC297008

[22] Vanholder R Schepers E Pletinck A Nagler EV Glorieux G. The uremic toxicity of indoxyl sulfate and p-cresyl sulfate: a systematic review. J Am Soc Nephrol (2014) 25(9):1897–907. 10.1681/ASN.2013101062 24812165 PMC4147984

[23] Massague J. TGFbeta signalling in context. Nat Rev Mol Cell Biol (2012) 13(10):616–30. 10.1038/nrm3434 22992590 PMC4027049

[24] Fine LG Hammerman MR Abboud HE. Evolving role of growth factors in the renal response to acute and chronic disease. J Am Soc Nephrol (1992) 2(7):1163–70. 1591357 10.1681/ASN.V271163

[25] Kaneto H Morrissey J Klahr S. Increased expression of TGF-beta 1 mRNA in the obstructed kidney of rats with unilateral ureteral ligation. Kidney Int (1993) 44(2):313–21. 10.1038/ki.1993.246 8377375

[26] Kopp JB Factor VM Mozes M Nagy P Sanderson N Bottinger EP Transgenic mice with increased plasma levels of TGF-beta 1 develop progressive renal disease. Lab Invest (1996) 74(6):991–1003. 8667617

[27] Lavoie P Robitaille G Agharazii M Ledbetter S Lebel M Lariviere R. Neutralization of transforming growth factor-beta attenuates hypertension and prevents renal injury in uremic rats. J Hypertens (2005) 23(10):1895–903. 10.1097/01.hjh.0000182521.44440.c5 16148614

[28] Motojima M Hosokawa A Yamato H Muraki T Yoshioka T. Uremic toxins of organic anions up-regulate PAI-1 expression by induction of NF-kappaB and free radical in proximal tubular cells. Kidney Int (2003) 63(5):1671–80. 10.1046/j.1523-1755.2003.00906.x 12675842

[29] Zhao YY Cheng XL Wei F Bai X Tan XJ Lin RC Intrarenal metabolomic investigation of chronic kidney disease and its TGF-beta1 mechanism in induced-adenine rats using UPLC Q-TOF/HSMS/MS(E). J Proteome Res (2013) 12(2):692–703. 10.1021/pr3007792 23227912

[30] Barreto FC Barreto DV Liabeuf S Meert N Glorieux G Temmar M Serum indoxyl sulfate is associated with vascular disease and mortality in chronic kidney disease patients. Clin J Am Soc Nephrol (2009) 4(10):1551–8. 10.2215/CJN.03980609 19696217 PMC2758258

[31] Adijiang A Goto S Uramoto S Nishijima F Niwa T. Indoxyl sulphate promotes aortic calcification with expression of osteoblast-specific proteins in hypertensive rats. Nephrol Dial Transplant (2008) 23(6):1892–901. 10.1093/ndt/gfm861 18334529

[32] Chiu CA Lu LF Yu TH Hung WC Chung FM Tsai IT Increased levels of total P-cresylsulphate and indoxyl sulphate are associated with coronary artery disease in patients with diabetic nephropathy. Rev Diabet Stud (2010) 7(4):275–84. 10.1900/RDS.2010.7.275 21713315 PMC3143542

[33] Miyazaki T Ise M Seo H Niwa T. Indoxyl sulfate increases the gene expressions of TGF-beta 1, TIMP-1 and pro-alpha 1(I) collagen in uremic rat kidneys. Kidney Int Suppl (1997) 62:S15–22. 9350672

[34] Miyazaki T Ise M Hirata M Endo K Ito Y Seo H Indoxyl sulfate stimulates renal synthesis of transforming growth factor-beta 1 and progression of renal failure. Kidney Int Suppl (1997) 63:S211–4. 9407462

[35] Kutz SM Hordines J McKeown-Longo PJ Higgins PJ. TGF-beta1-induced PAI-1 gene expression requires MEK activity and cell-to-substrate adhesion. J Cell Sci (2001) 114(Pt 21):3905–14. 11719557 10.1242/jcs.114.21.3905

[36] Pham BT van Haaften WT Oosterhuis D Nieken J de Graaf IA Olinga P. Precision-cut rat, mouse, and human intestinal slices as novel models for the early-onset of intestinal fibrosis. Physiol Rep (2015) 3(4):e12323. 10.14814/phy2.12323 25907784 PMC4425951

[37] Eddy AA Fogo AB Plasminogen activator inhibitor-1 in chronic kidney disease: evidence and mechanisms of action. J Am Soc Nephrol (2006) 17(11):2999–3012. 10.1681/ASN.2006050503 17035608

[38] Saito S Shimizu H Yisireyili M Nishijima F Enomoto A Niwa T. Indoxyl sulfate-induced activation of (pro)renin receptor is involved in expression of TGF-beta1 and alpha-smooth muscle actin in proximal tubular cells. Endocrinology (2014) 155(5):1899–907. 10.1210/en.2013-1937 24601883

[39] Sun CY Chang SC Wu MS. Uremic toxins induce kidney fibrosis by activating intrarenal renin-angiotensin-aldosterone system associated epithelial-to-mesenchymal transition. PLoS One (2012) 7(3):e34026. 10.1371/journal.pone.0034026 22479508 PMC3316590

[40] Galichon P Finianos S Hertig A. EMT-MET in renal disease: should we curb our enthusiasm? Cancer Lett (2013) 341(1):24–9. 10.1016/j.canlet.2013.04.018 23612071

[41] Xu-Dubois YC Baugey E Peltier J Colombat M Ouali N Jouanneau C Epithelial phenotypic changes are associated with a tubular active fibrogenic process in human renal grafts. Hum Pathol (2013) 44(7):1251–61. 10.1016/j.humpath.2012.10.010 23332931

[42] Bolati D Shimizu H Higashiyama Y Nishijima F Niwa T. Indoxyl sulfate induces epithelial-to-mesenchymal transition in rat kidneys and human proximal tubular cells. Am J Nephrol (2011) 34(4):318–23. 10.1159/000330852 21849772

[43] Kim SH Yu MA Ryu ES Jang YH Kang DH. Indoxyl sulfate-induced epithelial-to-mesenchymal transition and apoptosis of renal tubular cells as novel mechanisms of progression of renal disease. Lab Invest (2012) 92(4):488–98. 10.1038/labinvest.2011.194 22231736

[44] Mutsaers HA Engelke UF Wilmer MJ Wetzels JF Wevers RA van den Heuvel LP Optimized metabolomic approach to identify uremic solutes in plasma of stage 3-4 chronic kidney disease patients. PLoS One (2013) 8(8):e71199. 10.1371/journal.pone.0071199 23936492 PMC3732267

[45] Ferenbach DA Bonventre JV. Mechanisms of maladaptive repair after AKI leading to accelerated kidney ageing and CKD. Nat Rev Nephrol (2015) 11(5):264–76. 10.1038/nrneph.2015.3 25643664 PMC4412815

[46] Shimizu H Bolati D Adijiang A Enomoto A Nishijima F Dateki M Senescence and dysfunction of proximal tubular cells are associated with activated p53 expression by indoxyl sulfate. Am J Physiol Cell Physiol (2010) 299(5):C1110–7. 10.1152/ajpcell.00217.2010 20720180

[47] Shimizu H Yisireyili M Nishijima F Niwa T. Stat3 contributes to indoxyl sulfate-induced inflammatory and fibrotic gene expression and cellular senescence. Am J Nephrol (2012) 36(2):184–9. 10.1159/000341515 22889746

[48] Kuro-o M Matsumura Y Aizawa H Kawaguchi H Suga T Utsugi T Mutation of the mouse klotho gene leads to a syndrome resembling ageing. Nature (1997) 390(6655):45–51. 10.1038/36285 9363890

[49] Hu MC Shiizaki K Kuro-o M Moe OW. Fibroblast growth factor 23 and Klotho: physiology and pathophysiology of an endocrine network of mineral metabolism. Annu Rev Physiol (2013) 75:503–33. 10.1146/annurev-physiol-030212-183727 23398153 PMC3770142

[50] Mutsaers HA Levtchenko EN Martinerie L Pertijs JC Allegaert K Devriendt K Switch in FGFR3 and -4 expression profile during human renal development may account for transient hypercalcemia in patients with Sotos syndrome due to 5q35 microdeletions. J Clin Endocrinol Metab (2014) 99(7):E1361–7. 10.1210/jc.2014-1123 24670087 PMC5373678

[51] Doi S Zou Y Togao O Pastor JV John GB Wang L Klotho inhibits transforming growth factor-beta1 (TGF-beta1) signaling and suppresses renal fibrosis and cancer metastasis in mice. J Biol Chem (2011) 286(10):8655–65. 10.1074/jbc.M110.174037 21209102 PMC3048747

[52] Hu MC Kuro-o M Moe OW Klotho and chronic kidney disease. Contrib Nephrol (2013) 180:47–63. 10.1159/000346778 23652549 PMC3911771

[53] Adijiang A Shimizu H Higuchi Y Nishijima F Niwa T. Indoxyl sulfate reduces klotho expression and promotes senescence in the kidneys of hypertensive rats. J Ren Nutr (2011) 21(1):105–9. 10.1053/j.jrn.2010.10.020 21195930

[54] Sun CY Chang SC Wu MS. Suppression of Klotho expression by protein-bound uremic toxins is associated with increased DNA methyltransferase expression and DNA hypermethylation. Kidney Int (2012) 81(7):640–50. 10.1038/ki.2011.445 22237753 PMC3306006

[55] de Loor H Bammens B Evenepoel P De Preter V Verbeke K Gas chromatographic-mass spectrometric analysis for measurement of p-cresol and its conjugated metabolites in uremic and normal serum. Clin Chem (2005) 51(8):1535–8. 10.1373/clinchem.2005.050781 16040852

[56] Mutsaers HAM Caetano-Pinto P Seegers AEM Dankers ACA van den Broek PHH Wetzels JFM Proximal tubular efflux transporters involved in renal excretion of p-cresyl sulfate and p-cresyl glucuronide: implications for chronic kidney disease pathophysiology. Toxicol In Vitro (2015) 29(7):1868–77. 10.1016/j.tiv.2015.07.020 26216510

[57] Watanabe H Miyamoto Y Honda D Tanaka H Wu Q Endo M p-Cresyl sulfate causes renal tubular cell damage by inducing oxidative stress by activation of NADPH oxidase. Kidney Int (2013) 83(4):582–92. 10.1038/ki.2012.448 23325087

[58] Mutsaers HA van den Heuvel LP Ringens LH Dankers AC Russel FG Wetzels JF Uremic toxins inhibit transport by breast cancer resistance protein and multidrug resistance protein 4 at clinically relevant concentrations. PLoS One (2011) 6(4):e18438. 10.1371/journal.pone.0018438 21483698 PMC3070735

[59] Mutsaers HA Wilmer MJ Reijnders D Jansen J van den Broek PH Forkink M Uremic toxins inhibit renal metabolic capacity through interference with glucuronidation and mitochondrial respiration. Biochim Biophys Acta (2013) 1832(1):142–50. 10.1016/j.bbadis.2012.09.006 23017367

[60] Satoh M Hayashi H Watanabe M Ueda K Yamato H Yoshioka T Uremic toxins overload accelerates renal damage in a rat model of chronic renal failure. Nephron Exp Nephrol (2003) 95(3):e111–8. 10.1159/000074327 14646363

[61] Zhang Y Proenca R Maffei M Barone M Leopold L Friedman JM. Positional cloning of the mouse obese gene and its human homologue. Nature (1994) 372(6505):425–32. 10.1038/372425a0 7984236

[62] Meyer C Robson D Rackovsky N Nadkarni V Gerich J. Role of the kidney in human leptin metabolism. Am J Physiol (1997) 273(5 Pt 1):E903–7. 9374675 10.1152/ajpendo.1997.273.5.E903

[63] Shankar A Syamala S Xiao J Muntner P. Relationship between plasma leptin level and chronic kidney disease. Int J Nephrol (2012) 2012:269532. 10.1155/2012/269532 22666590 PMC3361181

[64] Sharma K Considine RV Michael B Dunn SR Weisberg LS Kurnik BR Plasma leptin is partly cleared by the kidney and is elevated in hemodialysis patients. Kidney Int (1997) 51(6):1980–5. 10.1038/ki.1997.269 9186891

[65] Alix PM Guebre-Egziabher F Soulage CO. Leptin as an uremic toxin: deleterious role of leptin in chronic kidney disease. Biochimie (2014) 105:12–21. 10.1016/j.biochi.2014.06.024 25010649

[66] Wolf G Hamann A Han DC Helmchen U Thaiss F Ziyadeh FN Leptin stimulates proliferation and TGF-beta expression in renal glomerular endothelial cells: potential role in glomerulosclerosis [see comments]. Kidney Int (1999) 56(3):860–72. 10.1046/j.1523-1755.1999.00626.x 10469355

[67] Briffa JF Grinfeld E Mathai ML Poronnik P McAinch AJ Hryciw DH. Acute leptin exposure reduces megalin expression and upregulates TGFbeta1 in cultured renal proximal tubule cells. Mol Cell Endocrinol (2015) 401:25–34. 10.1016/j.mce.2014.11.024 25478926

[68] Lee CI Guh JY Chen HC Lin KH Yang YL Hung WC Leptin and connective tissue growth factor in advanced glycation end-product-induced effects in NRK-49F cells. J Cell Biochem (2004) 93(5):940–50. 10.1002/jcb.20222 15389880

[69] Simonds SE Pryor JT Ravussin E Greenway FL Dileone R Allen AM Leptin mediates the increase in blood pressure associated with obesity. Cell (2014) 159(6):1404–16. 10.1016/j.cell.2014.10.058 25480301 PMC4259491

[70] Allison MA Ix JH Morgan C McClelland RL Rifkin D Shimbo D Higher leptin is associated with hypertension: the Multi-Ethnic Study of Atherosclerosis. J Hum Hypertens (2013) 27(10):617–22. 10.1038/jhh.2013.24 23535989 PMC3735864

[71] Schepers E Speer T Bode-Boger SM Fliser D Kielstein JT. Dimethylarginines ADMA and SDMA: the real water-soluble small toxins? Semin Nephrol (2014) 34(2):97–105. 10.1016/j.semnephrol.2014.02.003 24780466

[72] Mallipattu SK He JC Uribarri J. Role of advanced glycation endproducts and potential therapeutic interventions in dialysis patients. Semin Dial (2012) 25(5):529–38. 10.1111/j.1525-139X.2012.01081.x 22548330 PMC5558608

[73] Bagrov AY Shapiro JI Fedorova OV. Endogenous cardiotonic steroids: physiology, pharmacology, and novel therapeutic targets. Pharmacol Rev (2009) 61(1):9–38. 10.1124/pr.108.000711 19325075 PMC2763610

[74] Gonick HC Ding Y Vaziri ND Bagrov AY Fedorova OV. Simultaneous measurement of marinobufagenin, ouabain, and hypertension-associated protein in various disease states. Clin Exp Hypertens (1998) 20(5–6):617–27. 10.3109/10641969809053240 9682918

[75] Komiyama Y Dong XH Nishimura N Masaki H Yoshika M Masuda M A novel endogenous digitalis, telocinobufagin, exhibits elevated plasma levels in patients with terminal renal failure. Clin Biochem (2005) 38(1):36–45. 10.1016/j.clinbiochem.2004.08.005 15607315

[76] Fedorova LV Raju V El-Okdi N Shidyak A Kennedy DJ Vetteth S The cardiotonic steroid hormone marinobufagenin induces renal fibrosis: implication of epithelial-to-mesenchymal transition. Am J Physiol Renal Physiol (2009) 296(4):F922–34. 10.1152/ajprenal.90605.2008 19176701 PMC3973402

[77] Haller ST Drummond CA Yan Y Liu J Tian J Malhotra D Passive immunization against marinobufagenin attenuates renal fibrosis and improves renal function in experimental renal disease. Am J Hypertens (2014) 27(4):603–9. 10.1093/ajh/hpt169 24014658 PMC3958603

[78] Lekawanvijit S Kompa AR Manabe M Wang BH Langham RG Nishijima F Chronic kidney disease-induced cardiac fibrosis is ameliorated by reducing circulating levels of a non-dialysable uremic toxin, indoxyl sulfate. PLoS One (2012) 7(7):e41281. 10.1371/journal.pone.0041281 22829936 PMC3400638

[79] Bolati D Shimizu H Niwa T. AST-120 ameliorates epithelial-to-mesenchymal transition and interstitial fibrosis in the kidneys of chronic kidney disease rats. J Ren Nutr (2012) 22(1):176–80. 10.1053/j.jrn.2011.10.015 22200438

[80] Evenepoel P Meijers BK Bammens BR Verbeke K. Uremic toxins originating from colonic microbial metabolism. Kidney Int Suppl (2009) 114:S12–9. 10.1038/ki.2009.402 19946322

[81] Masereeuw R Mutsaers HA Toyohara T Abe T Jhawar S Sweet DH The kidney and uremic toxin removal: glomerulus or tubule? Semin Nephrol (2014) 34(2):191–208. 10.1016/j.semnephrol.2014.02.010 24780473

